# Editorial

**Published:** 1899-05

**Authors:** 


### HOMŒOPATHIC PHYSICIAN,

A MONTHLY JOURNAL OF

HOMŒOPATHIC MATERIA MEDICA AND CLINICAL MEDICINE.

If our school ever gives up the strict inductive method of Hahnemann, we are lost, and deserve only to be mentioned as a caricature in the history of medicine."—CONSTANTINE HERING.

Vol. XIX. MAY, 1899. No. 5.

### EDITORIAL.

THE PRESIDENT OF THE INTERNATIONAL HAHNEMANNIAN ASSOCIATION makes a final appeal to the members to use their utmost efforts to attend the next meeting in June (27th) at the International Hotel, Niagara Falls.

The past winter has been so full of remakable occurrences in the medical world that an interesting accumulation of experience must have fallen to the share of every member. The free interchange of these experiences must be in the highest degree useful to every one. The comparison of results and the clearing up of misapprehensions or mistaken views either upon the principle of Homœopathy itself or upon the sphere and scope of the remedies and the significance and value of symptoms or their genuineness, most instructive.

The knowledge thus acquired is of the highest value in treating cases met with in our every-day life.

It would seem superfluous to recount all this to homœopathists, as they certainly must know it well. But we find that they do overlook it and consequently are somewhat indifferent to the need of attending the meetings, or of communicating papers that are beneficial to the majority.

Some who attend may be found listening to the voice of intrigue that seeks some selfish advancement at the expense of some other member or members, and with the grave result of irretrievably damaging the cause and disintegrating the society. Those who do allow these intrigues to command their attention are belittling themselves, and taking their minds off the great object of the meeting, and assisting in the perpetration of evil, while they inevitably contribute to the dwarfing of the proceedings and bringing them perilously near to total failure.

Experience in the past should be recalled and a lesson taken to carefully avoid similar occurrences in the future.

